# Nutritional composition of edible wood borer beetle larvae in Kenya

**DOI:** 10.1371/journal.pone.0304944

**Published:** 2024-06-06

**Authors:** Shadrack Kibet, Cynthia M. Mudalungu, Brian O. Ochieng, Hosea O. Mokaya, Njogu M. Kimani, Chrysantus M. Tanga

**Affiliations:** 1 International Centre of Insect Physiology and Ecology (*icipe*), Nairobi, Kenya; 2 Department of Physical Sciences, University of Embu, Embu, Kenya; 3 School of Chemistry and Material Science, The Technical University of Kenya, Nairobi, Kenya; Institute for Biological Research, University of Belgrade, SERBIA

## Abstract

Exploration of edible insects as sustainable alternative nutrient-dense sources such as nutraceuticals have attracted more and more global attention recently. However, research on wood borer beetles have largely been overlooked. This study assessed the entomo-chemical properties of *Titoceres jaspideus* (Cerambycidae) and *Passalus punctiger* (Passalidae), which are widely consumed in many African countries, including Kenya. The crude protein content of the beetle larvae ranged between 27.5–39.8 mg BSA/g. In comparison with those of cereals, amino acids such as lysine (7.9–9.9 mg/g), methionine (0.48–0.64 mg/g) and threonine (2.31–2.55 mg/g) were considerably high in the larvae. Methyl-5*Z*,8*Z*,11*Z*,14*Z*-eicosatetraenoate and methyl-9*Z*-octadecenoate were the predominant polyunsaturated and monounsaturated fatty acids, respectively. High total phenols (>4.4 mg GAE/g), flavonoids (>3.6 mg QE/g) and anti-oxidative activities (>67%) were recorded for both larvae. This implies that increasing the consumption of wood-borer beetle larvae would positively impact the state of the natural environment and reduce the problem of malnutrition in the society. Thus, applying these strategies to develop insect food in a more familiar form can help to make insect-enriched foods more appealing to consumers, facilitating their widespread adoption as a sustainable and nutritious food source.

## Introduction

Malnutrition is one of the most severe worldwide challenges affecting approximately a billion people [1]. This number is unfortunately expected to rise as the world’s population surges ahead with estimates that it is likely to hit the 9.6 billion mark by 2050 and therefore food demands may not be met [2]. The food production systems currently relied on are no longer sustainable due to the diminution of arable lands [3]. Further, they have been associated with negative environmental and health effects such as the emission of at least 20% of total greenhouse gases by ruminants [4] and the transmission of bovine zoonotic diseases [5]. The over-reliance on red meat as the main source of protein by humans has lately been linked to cancers such as breast, endometrial, colorectal, colon, rectal, lung, and hepatocellular carcinoma [6, 7]. The obstacles arising from livestock farming and the consumption of animal products, combined with the United Nation’s approaching deadlines for achieving zero hunger [8], underscore the urgent necessity for dietary diversification. Alternative foods to minimize the current challenges are sought after.

Entomophagy, the eating of insects has long been practised globally throughout human history. While it has been a long-standing tradition in African, Latin American and Asian cultures, its popularity is now spreading to Europe and North America [9, 10]. This shift suggests a broader recognition of insects as a viable and eco-friendly food choice across various regions and societies. There are more than 2000 edible insect species from the orders Coleoptera, Hymenoptera, Orthoptera, Lepidoptera, Hemiptera, Diptera, Odonata and Blattodea [11, 12]. Edible insects are ranked as sustainable food sources for various reasons including less land and water utilization to rear them, emission of less greenhouse gases, high fecundity, high feed conversion efficiencies, high edible body mass percentage, and high nutritional value [13–16]. Despite their vast biodiversity and distribution, it is documented that only a few have been commercialized [17] and about 2% of all the known edible insects have established rearing units [18].

Researchers have recently shifted their attention to insects as a potential source of nutrition due to their high nutrient density, abundance, sustainability and efficiency in feed conversion. Their protein content ranges from 20–70% dry weight (dw) depending on the insect species, developmental stage and diet while their fat content is estimated to be 2–50% dw [14], majorly comprising of unsaturated fatty acids. Furthermore, they are rich in phytochemicals such as flavonoids and phenols that serve as free radical scavengers [19]. For instance, a recent study conducted in Colorado demonstrated that consuming breakfast supplemented with 25 g of cricket powder for two weeks can lead to a significant reduction in plasma levels of TNF-α (i.e., a pro-inflammatory cytokine), with potential to mitigate systemic inflammation [20]. It has also been demonstrated that hydrolysates derived from *Tenebrio molitor* larvae possess the ability to scavenge free radicals while *Schistocerca gregaria* protein hydrolysates exhibits a high potential for lipoxygenase and cyclooxygenase-2 inhibitory activities [21]. Insects and their gut symbionts are also a source of bioactive natural products with enormous potential for use in the fight against drug resistant pathogens [22]. Furthermore, chitin derived from crickets offers a potential prebiotic solution for naturing probiotic microbes in animal and human gut [23].

Xylophagous beetle larvae feed on wood, but have been relatively understudied in terms of their nutritional content despite their traditional use as a food source in many countries [24] including North-East Indian tribal societies [25]. Additionally, huhu grubs belonging to the family Cerambycidae has been regularly featured at the New Zealand’s food festival [26] as delicacies.

Previous studies on *Thysia wallichii*, a semi-domesticated wood borer in Nagaland, revealed that it contains 56.18 ± 4.06% protein and 5.51 ± 0.07% lipid [27]. Studies on *Prionoplus reticularis*, a xylopagous species native to New Zealand, indicated that they possess a unique fatty acid profile, characterized by relatively low levels of saturated fatty acids (SFA) and high levels of monounsaturated fatty acids (MUFA) compared with commonly consumed sources in eggs and beef [26]. These fatty acids composition suggests that these beetles may have a positive impact on human health, yet other species of these beetles remain understudied, particularly in Africa. The objective of the current study was to assess the protein, fatty acid and amino acid compositions as well as entomo-chemical properties (total flavonoids and phenolic contents, and antioxidant properties) of two edible xylophagous beetles (*Passalus punctiger* and *Titoceres jaspideus*) found in two different forested areas of Kenya.

## Materials and methods

### Sampling sites

Wood-borer beetle larvae were collected from Kakamega forest (0°17’ 18.00" N, 34° 51’ 13.19" E) and Mau forest (0.6096° S, 35.7354° E). A forest access permit (RESEA/1/KFS/VOL.V11-37) was provided by the Kenya Forest Service. Kakamega forest is a mid-altitude (1500-1600m) tropical rainforest located in two Kenyan counties; Kakamega and Nandi. It is the only remnant of the ancient Eastern patch of the Guineo-Congolian rainforest that once spanned Kenya, Zaire and Uganda [28]. The forest temperature ranges 20–30°C and receives both long rains (April and May) and short rains (August and September), annually averaging between 1200–1700 mm. It serves as a source of Rivers Isiukhu and Yala that both drains to Lake Victoria. The forest is an important and unique hotspot for diverse flora and fauna, inclusive of species that are rare in other parts of Kenya [28].

Mau forest is the largest indigenous montane forest in Eastern Africa, situated in four Kenyan counties: Narok, Nakuru, Bomet and Kericho, at an elevation range of 1800–3000 m. The annual rainfall and temperature ranges between 1000–2000 mm and 16–24°C, respectively. Despite the long term effect of human activities such as logging, charcoal burning, human habitation and hunting, the forest still remains a biodiversity avenue for a variety of tree species including *Croton megalocarpus*, *Teclea nobilis*, *Juniperus procera*, *Hagenia abyssinica*, *Allophylus abyssinica* among others [29].

### Sample collection

Sampling was conducted by the first author of this manuscript in collaboration with personnel from the Kenya Forest Service and local community members, from May 27^th^ to June 5^th^, 2022, during daytime hours. In each forest, three accessible sites were visited and a total of 400 fallen-rotten or decomposing wood of suitable host plants were randomly sampled per site within the forested areas. A systematic approach was employed to identify and select specific rotting wood known for their propensity to host beetle larvae. These were: *Prunus aficana*, *C*. *megalocarpus*, *Harungana madagascariensis*, *Bridelia micrantha*, *Polyscias fulva*, *maesopsis eminii* and *Croton macrostachyus*. Initial surveys involved visual inspection of the forest floors to locate fallen logs exhibiting intermediate or advanced stages of decomposition. The logs were further screened by gentle probing, peeling the barks and through visual observation of galleries and exit holes. Positively selected host woods were identified by a botanist (Mr. Moses Livasia) provided by the Kenya Forest Service. The larvae and adults were collected upon splitting the dead woods using an axe, ice-packed in cool boxes and transported to the laboratory at the International Centre of Insect Physiology and Ecology (*icipe)*, Nairobi. Samples were frozen at -80°C until used for further experimental processes.

### Molecular identification of beetles

The frozen adult beetles, one each, were left to thaw before carrying out DNA extraction. To eliminate PCR-inhibitors, the insects were first washed using tap water, surface sterilized using 70% ethanol and subsequently rinsed three times with sterilized distilled water. The insect legs were separated from the body using a sterile scalpel blade, placed in a micro-centrifuge tube and later on ground in liquid nitrogen. The homogenate was subjected to DNA extraction using Isolate II genomic DNA extraction kit (Bioline) keenly following manufacturer’s instructions. To identify the beetles, a 20 *μ*L Polymerase chain reaction (PCR) was carried out. The reaction mixture contained 4 *μ*L HOT FIREPol® Blend Master Mix (Solis BioDyne, Tartu, Estonia), 1.0 *μ*L of 10 *μ*M reverse and forward primers, 4 *μ*L of the template DNA, and 10 *μ*L of nuclease free water was performed on ProFlex PCR system thermocycler. The primers LCO1490 (5’-GGTCAACAAATCATAAAGATATTGG-3’) and HCO2198 (5’-TAAACTTCAGGGTGACCAAAAAATCA-3’) that target the cytochrome oxidase subunit 1 (CO 1) were used. The thermal cycling conditions were set as follows: initial denaturation at 95°C for 15 min followed by 35 cycles of denaturation at 95°C for 45 s, annealing at 53°C for 40 s and extension at 72°C for 45 s. The final extension was done at 72°C for 5 min and amplicons kept at infinite hold at 4°C. The PCR products were analyzed by running on 2% agarose gel at 100V for 1 h before visualization on UV trans-illuminator. The positive amplicons were cleaned using ExoSap^TM^ according to manufacturer’s instruction and the purified products sent for Sanger sequencing at Macrogen Inc. (Amsterdam, Nertherlands). The sequences were analyzed by trimming, editing and aligning using the Geneious Prime Software (Biomatter Ltd., Auckland, New Zealand) v2023.0.3 [30]. The aligned sequences were extracted and queried against the GenBank database using the Basic Local Alignment Search Tool (BLASTn) to identify similar matches.

### Nutritional analyses

#### Protein determination

From each collection site, at least 20 oven dried (SDO-225, Wagtech International, Thatcham, UK at 60°C for 24 h) larvae were ground in a three-speed Waring laboratory blender (Camlab, Over, UK) to fine powder. The total protein content was determined according to the Bradford method [31] with minor modifications. Ground insect samples (10 mg) were suspended in 2 mL distilled water and allowed to rest for 2 h, with mild agitations at intervals of 30 min. The suspensions were centrifuged at 10000 rpm (Eppendorf^®^ 5430) for 10 min, and 200 *μ*L of the supernatants transferred into clean 15 mL falcon tubes. Into each of the falcon tubes, 5 mL of pre-prepared Coomassie Brilliant Blue reagent was added and after 2 min, absorbance was measured at 595 nm against a blank using a UV-Vis spectrophotometer (Evolution Pro- Thermo scientific). Bovine serum albumin (BSA) standards (15.6–1000 *μ*g/mL), used to generate a calibration curve, were prepared the same way as the samples, and their absorbance recorded at 595 nm. The total protein content was calculated and expressed as milligrams (mg) of BSA equivalents/g dw.

#### Amino acid analysis

Amino acids profiling was conducted according to the protocols described by Murugu et al. (2021) [32] with slight modifications. Dried insect powders (100 mg) were hydrolyzed in 1.5 mL of 6N HCl at 110°C for 24 h under nitrogen. The hydrolysates were then evaporated at 40°C to dryness in a stream of nitrogen and the residues reconstituted in 1 mL of 0.01% formic acid/acetonitrile (95:5). The mixture was vortexed for 30 s, sonicated for 30 min, and then centrifuged at 14,000 rpm. The supernatant was filtered through a 0.45 *μ*m syringe filter and analyzed on an Agilent 1290 HPLC coupled to a 6120 series single quad MS Agilent (Technologies Inc.,Santa Clara, CA, USA). The chromatographic separation was achieved using a Zorbax RX-C18, 4.6 × 250mm, 5 *μ*m column, operated at 40°C. The mobile phase comprised of water (A) and acetonitrile (B) all supplemented with + 0.01% formic acid. The gradient elution adopted was as follows: 0–6 min, 10% B; 6–7.5 min, 10–80% B; 7.5–10.5 min, 80% B; 10.5–13 min, 80–100% B; 13–18 min, 100% B; 18–20 min, 100–10% B; 20–25 min, 10% B. The flow rate was programmed as follows: 0–13 min; 0.25 mL/min, 13–25; 0.5 mL/min and the injection volume was 5 *μ*L. The mass spectrometer was operated on API-ES positive mode at a mass range of m/z 50–600 at 70 eV cone voltage. An authentic standard of amino acids (Sigma–Aldrich, St. Louis, MO, USA) was analyzed by LC-MS and used to externally quantify the amino acids. All the analyses were performed in triplicates.

#### Fatty acid analysis

Extraction of total lipids, methylation and analysis of fatty acids were determined following methods described by Ochieng et al. [33]. Briefly, 1 g of the samples were subjected to a Folch-based extraction with 10 mL of dichloromethane/methanol (2:1 v/v) containing 0.05 mg/mL butylated hydroxytoluene (BHT). Upon centrifugation at 4200 rpm for 10 min, the supernatants were evaporated under vacuum to recover oils of approximately 300 mg. Oils (100 mg each) were methylated by introducing 1 mL of sodium methoxide solution (100 mg/mL), vortexing for 1 min, sonicating for 10 min and incubating in a water bath at 70°C for 1 h. The reaction was halted by adding 100 *μ*L of distilled deionized water and vortexing for another 1 min. The resulting fatty acids were extracted with 1 mL of gas chromatography (GC)-grade hexane (Sigma–Aldrich, St. Louis, MO, USA) and the extracts centrifuged for 20 min at 14,000 rpm. An aliquot (1.0 *μ*L) of the supernatants, carefully dried over anhydrous sodium sulphate and filtered, was analyzed by GC-MS on a 7890A gas chromatograph linked to a 5975C mass selective detector (Agilent Technologies Inc.,Santa Clara, CA, USA). The GC was equipped with a (5%-phenyl)-methylpolysiloxane (HP-5MS) low bleed capillary column (30 m × 0.25 mm i.d., 0.25 *μ*m; J&W, Folsom, CA, USA). Helium at a flow rate of 1.25 mL/min was the carrier gas. At a rising rate of 10°C/ min, the oven temperature was programmed from 35°C to 285°C, with both the initial and final temperatures maintained for 5 min and 20.4 min, respectively. Both the ion source and quadrupole mass selective detector temperatures were maintained at 230°C and 180°C, respectively. The spectra from electron impact (EI) were acquired at an acceleration energy of 70 eV. The fragment ions were analyzed over 40–550 *m/z* mass range in the full scan mode and the filament delay time set at 3.3 min. Octadecanoic acid (≥95% purity) (Sigma-Aldrich, St. Louis, MO) was used to prepare serial dilutions of authentic standard methyl octadecanoate (0.2–125 ng/*μ*L). These dilutions were used to generate a linear calibration curve (peak area vs. concentration), yielding the equation; [y = 5E+7x+2E+7, R^2^ = 0.9997] and used for external quantification of the various fatty acids. ChemStation B.02.02 software was used for the data acquisition and the compounds identified by comparison of mass spectral data and retention times with those of authentic standards and reference spectra published by library MS databases: National Institute of Standards and Technology (NIST) 05, 08, and 11. Determination of the FAMEs was done in triplicates.

#### Determination of total flavonoids and phenols

The total flavonoids were determined using the aluminium chloride calorimetric assay [34], with slight modifications. Ground insect material (50 mg) was mixed with 3 mL of 50% methanol and allowed to rest for 2 h with a 30-min periodic agitation. Upon centrifugation of the suspension, 200 *μ*L of the supernatant was transferred into a clean test tube into which 300 *μ*L of 5% sodium nitrite were subsequently added. The mixture was allowed to rest for 5 min after which 300 *μ*L of 10% aluminium chloride was added. After 1 min, 2 mL of 1M sodium hydroxide and 2.4 mL of 50% methanol were added. Absorbance were recorded at 510 nm against a blank (50% methanol) using a UV-Vis spectrophotometer. Standards (Quercetin; 0.5 mg/mL, 1 mg/mL, 2 mg/mL and 8 mg/mL), prepared similarly, were also analyzed to generate a calibration curve for external quantification. The total flavonoid contents were expressed as milligram quercetin equivalents per gram of sample (mg QE/g), conversion factor = x60.

The total phenols were quantified calorimetrically using the Folin Ciocalteu reagent, following the published protocols [35] with minor modifications. The methanolic supernatant (200 *μ*L) was mixed with 1 mL of 0.2 N Folin-Ciocalteu reagent and incubated at room temperature for 5 min after which 800 *μ*L of sodium carbonate (75 g/L) was added. The mixtures were incubated for 2 h at room temperature after which their absorbance were measured at 760 nm against a reagent blank using a UV-Vis spectrophotometer. Similarly, standards of authentic Gallic acid (1 mg/mL and its two-fold serial dilutions) were prepared and analyzed for generation of a calibration curve. The total phenols were expressed as milligram Gallic acid equivalents per gram of sample (mg GAE/g dw), conversion factor = x60.

#### Determination of antioxidant activity

Insect samples (5 mg) were soaked in 2 mL of methanol and incubated for 2 h at room temperature with periodical agitation. The suspensions were centrifuged at 4200 rpm for 10 min and the supernatants (750 *μ*L) transferred into clean test tubes. Exactly 1.5 mL of DPPH solution (0.05 mg/mL) was then added into each tube and the contents incubated for 10 min in the dark. The samples and control absorbance were measured at 517 nm against a blank. The blank was made up of 2 mL of methanol while the control comprised of 750 *μ*L of methanol and 1.5 mL of 0.05 mg/mL DPPH. The free radical scavenging activity of the insect powders were expressed as percentage inhibition using Eq 1.


$$\%\;Inhibition=\frac{Control\;absorbance\;(A0)-Sample\;absorbance\;(As)}{Control\;absorbance\;(A0)}\;X\;100\%$$ (1)

#### Data analysis

All experiments were carried out in triplicates and Bartlett’s test was employed to determine the homogeneity of variances. One-way ANOVA was adopted to establish significant difference among groups. Significant means were separated by Tukey’s post-hoc at α = 0.05. All statistical analyses were performed in R version 3.1.4 (R Core Team, 2022) [36] whereas graphs were generated using GraphPad software version 8.0.1 (San Diego, California USA).

## Results and discussions

### Molecular identification of beetles

**Fig 1:**
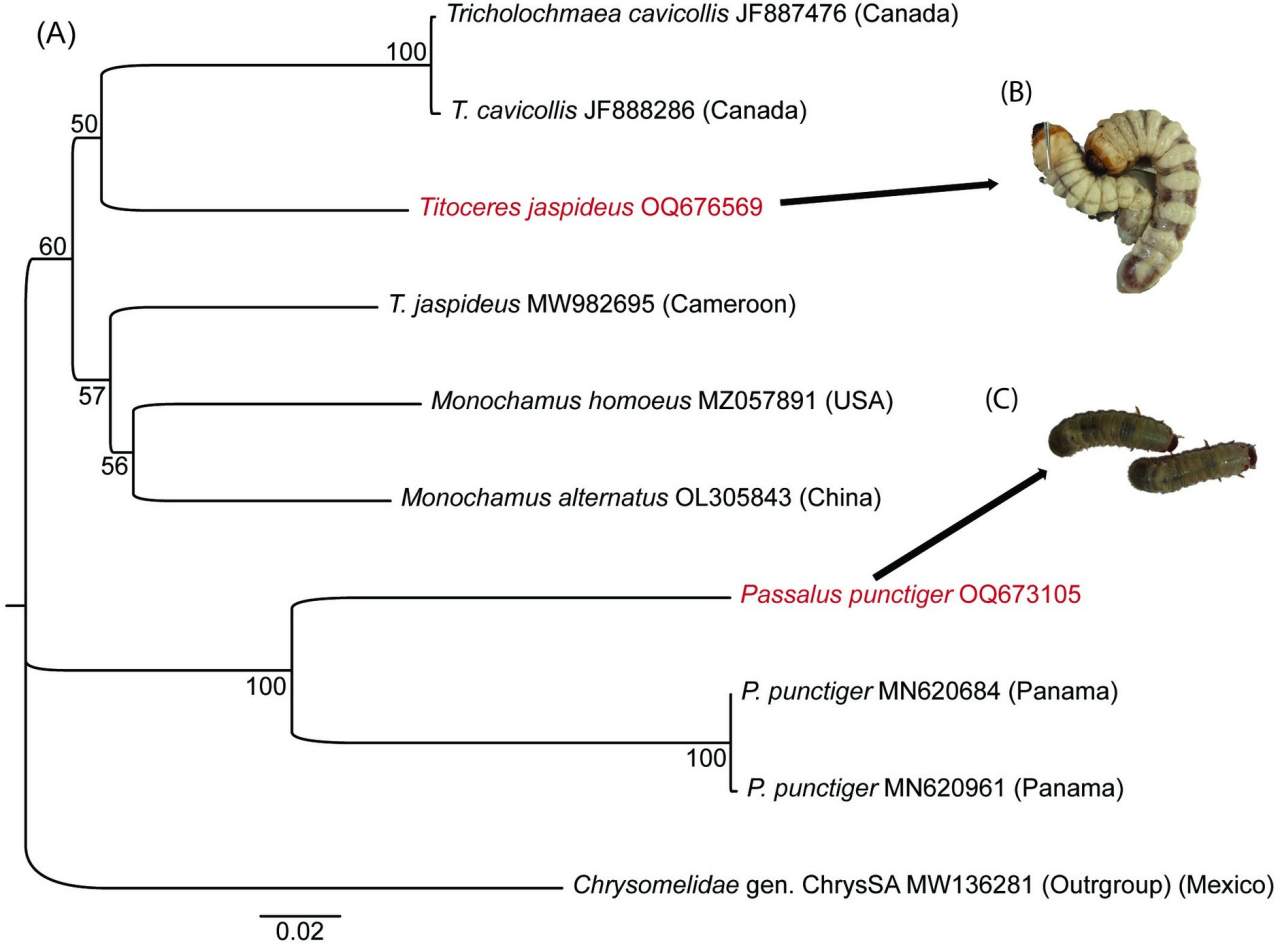
Neighbour-joining tree based on CO1 gene sequences showing *P*. *punctiger* and *T*. *jaspideus* alongside related species. (A). The numbers at the nodes are bootstrap values. *Chrysomelidae* (GenBank accession number MW136281) was used as an outgroup. (B) and (C) are photos of *T*. *jaspideus* and *P*. *punctiger* larvae, respectively.

The two beetles identified included *Passalus punctiger* Lepeletier, Serville [accession number OQ673105] and *Titoceres jaspideus* Audinet Serville [accession number OQ676569] (Fig 1A). The sequences of the two beetles were distinct from those in the GenBank database with the same names. The *P*. *punctiger* from this study had 81.80% identity match with the same identified organism in GenBank of accession number MN620684.1 from Panama. Contemporarily, *T*. *jaspideus* had 85.93% identity match with GenBank’s MW982695.1 from Cameroon. The dissimilarities in the sequences from this study and those from the database could be due to misidentification in the former submissions. Additional studies are paramount to resolve the sequence mismatching conflict.

The beetle *P*. *punctiger*, belongs to the family Passalidae of the superfamily Scarabaeoidea. Adult passalid has a body length range of 30–40 mm, while their larvae can reach a length of about 55 mm (Fig 1C). Freshly emerged adults are red-brown and turns black as they age [37]. On the other hand, *T*. *jaspideus* are long-horned beetles in the family Cerambycidae. The adults have long antennae (half their body lengths), dark-brown in colour and can grow to about 45 mm long. Their larvae are white, legless grubs that live within the dead, decomposing logs of indigenous trees and can be as long as 70 mm (Fig 1B) [38]. In Kakamega forest, the beetles inhabited decomposing logs of *Prunus africana*, *Croton megalocarpus*, *Harungana madagascariensis*, *Bridelia micrantha*, *Polyscias fulva* and *Maesopsis eminii* tree species while in Mau forest, they inhabited *C*. *megalocarpus* and *Croton macrostachyus* dead woods. In Kakamega forest, the total number of *T*. *jaspideus* larvae collected from the decomposing logs were: 107 (*P*. *africana*), 158 (*C*. *megalocarpus*), 72 (*H*. *madagascariensis*), 97 (*B*. *micrantha*), 95 (*P*. *fulva*), and 68 (*M*. *eminii*); whereas for *P*. *punctiger*, the counts were 74 (*P*. *africana*), 90 (*C*. *megalocarpus*), 72 (*H*. *madagascariensis*), 122 (*B*. *micrantha*), 95 (*P*. *fulva*), and 68 (*M*. *eminii*). In Mau forest *T*. *jaspideus* larvae counts from *C*. *megalocarpus* and *C*. *macrostachyus* were 81 and 70 respectively, while for *P*. *punctiger*, counts were 73 and 70 from the respective tree species. It was noted that, *T*. *jaspideus* appeared to prefer dead logs of *C*. *megalocarpus* while *P*. *punctiger* preferred *B*. *micrantha*.

### Nutritional analyses

#### Total protein content

**Fig 2:**
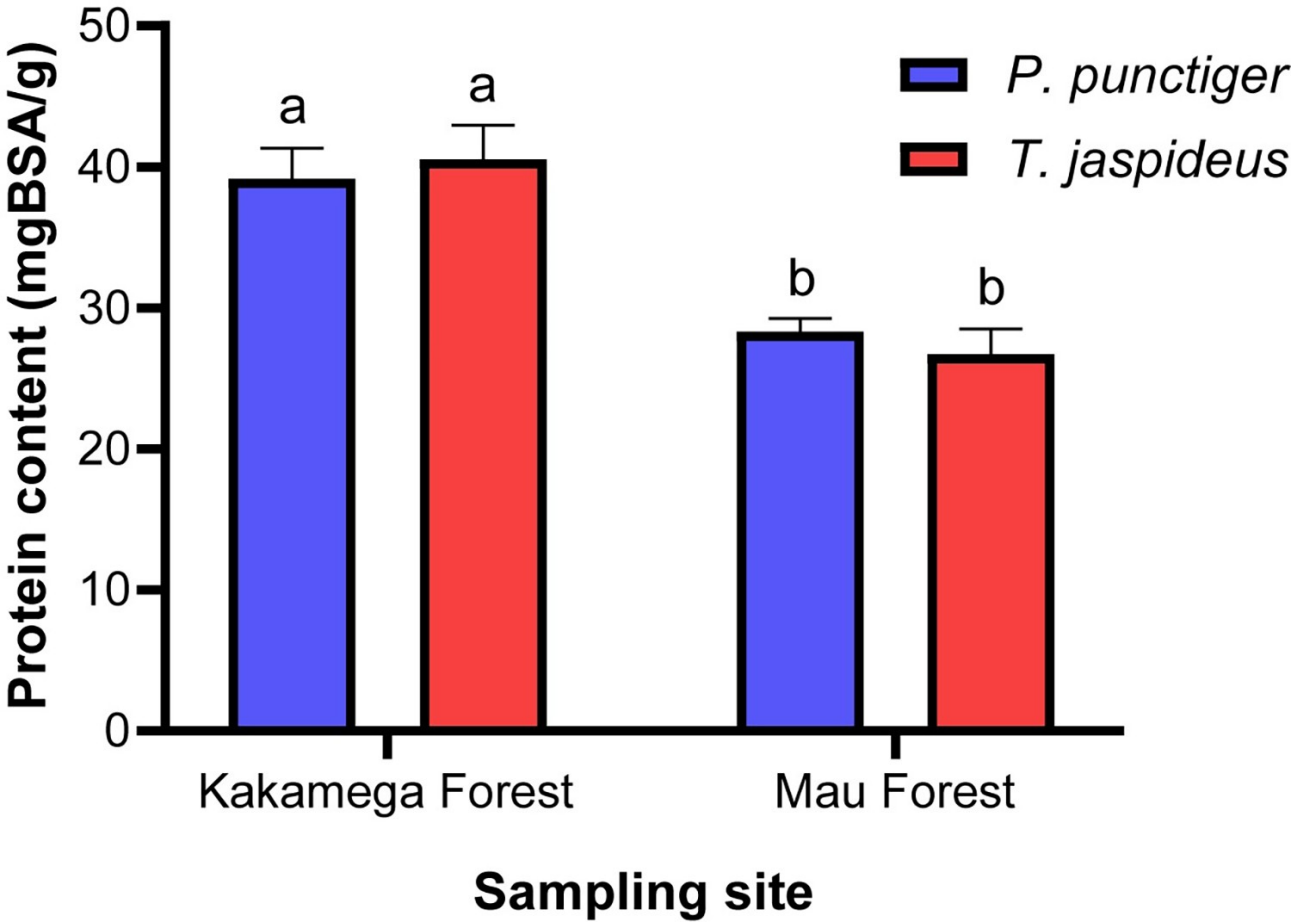
Protein content (mg BSA/g dw) of *P*. *punctiger* and *T*. *jaspideus*. Values are means ± standard deviation of replicates. Different letters above every bar indicate significant differences (*p*< 0.05).

The larvae of the two beetles, *T*. *jaspideus* (40.53 mg BSA/g dw) and *P*. *punctiger* (39.16 mg BSA/g dw) sourced from Kakamega forest exhibited significantly higher protein content (*p*<0.05) than their respective Mau forest counterpart species (Fig 2). The between sites dissimilarities in protein contents may be attributed to the differences in characteristic dietary sources, climatic condition, and geographical location of the two forests [39]. The protein levels of the two species from each forest were similar, further reinforcing the assertion on the effect of the two forest characteristics. That notwithstanding, the protein values were within the ranges of 34.1 to 78.9 mg BSAE/g as reported by Mokaya et al. [40] on domesticated and wild silk moths which has been widely studied and documented in literature.

The protein content of both beetle larvae reported herein may have been underestimated, which is a particular shortcoming when Bradford method is used [41], exposing its unreliability in protein estimation. Regardless, the current study proceeded to employ the method for protein estimation because it was the only available and dependable option at the study institution during data collection. This necessitates for more research to accurately quantify the protein contents of these beetle larvae using more sensitive approaches such as Kjeldahl method in the future. Nevertheless, this study provides new insights into the protein content of the two xylophagous insects and therefore popularizing the consumption of these lesser-utilized protein sources, with potential to serve as a more affordable alternative compared to costly animal protein sources.

#### Amino acid profile

The high protein content possessed by insects makes them rich sources of essential amino acids whose concentrations and composition vary with insect species, sampling location, developmental stages, food fed on by the insect, harvesting season and methods of sample preparation and analysis [42]. In the current study, a total of 14 amino acids were detected, comprising of 8 essential amino acids (EAA) and 6 non-essential amino acids (NEAA) (**Table 1**). Valine, lysine and leucine of the EAA and proline and glycine of the NEAA were the dominant amino acids, with no evidence of significant variation between species and between collection sites (*p*>0.05). The abundance of valine, lysine and leucine have previously been reported in edible beetle *Holotrichia parallela* [43], edible dung beetle larvae [44], and *Oryctes monoceros* and *Orycte boas* larvae [45]. The detection of considerable levels of lysine and threonine in the beetle larvae species demonstrates their possible nutritional superiority over plant-derived products, characterized with deficiency in such amino acids [14]. This study therefore provides insights into possible utilization of such potential novel food sources to help curb malnutrition through enrichment of commonly consumed cereal-based diets. Furthermore, it reveals methionine as the least abundant amino acid coupled with a non-detection of cysteine. Low or lack of sulphur containing amino acids has widely been reported in some edible insects [46], however limiting amino acids in insects depend on species and dietary source [47, 48]. In contrast to previous studies on other insects [49–51] and wood burrowing beetle *Anaplophora chinensis* [42], reporting predominating levels of glutamic acid over glycine, this study revealed contrasting results, with significantly higher levels of glycine compared to glutamic acid. In light of these findings, we hypothesize that the study insect may possess metabolic pathways tailored to elevate glycine levels, or possibly their diet contained more glycine than glutamic acid. Insignificant differences in the amino acid concentrations of *T*. *jaspideus* and *P*. *punctiger* from both Kakamega and Mau forests suggest that species and habitat differences did not influence their levels. This endorses earlier reports noting the independence of amino acids concentrations of insect species on dietary sources, with variabilities only emerging from differences in life stages or functional requirements of specific body parts [52]. Appreciable levels of aromatic amino acids (phenylalanine and tyrosine) from the two beetle species were also detected. These amino acids serve as precursors for the synthesis of many biologically/neurologically active compounds that are essential for maintaining normal biological functions. However, cysteine and tryptophan were not detected. Cysteine levels may have been interfered with by methionine during hydrolyses since inhibitive performic acid oxidation step was not performed [53, 54]. On the other hand, tryptophan was not found due to their possible destruction during acid hydrolysis and were not quantified by basic hydrolysis of the samples.

**Table 1:** Concentration of amino acids (mg/g) of *P*. *punctiger* and *T*. *jaspideus*.

	Kakamega forest		Mau forest			
Amino acids	*punctiger*	*T*. *jaspideus*	*P*. *punctiger*	*T*. *jaspideus*	F_(3,8)_-value	*p*-value
Valine	15.39 ± 0.94^a^	14.70± 3.86^a^	16.00 ± 0.01^a^	15.02 ± 1.11^a^	0.22	0.879
Threonine	2.55 ± 0.26^a^	2.40 ± 0.29^a^	2.31 ± 0.11^a^	2.43 ± 0.32^a^	0.46	0.721
Phenylalanine	6.74 ± 0.42^a^	6.13 ± 1.03^a^	6.68 ± 0.08^a^	7.09 ± 0.38^a^	1.34	0.327
Methionine	0.64 ± 0.04^a^	0.48 ± 0.06^a^	0.49 ± 0.04^a^	0.63 ± 0.13^a^	3.85	0.057
Lysine	8.56 ± 0.87^a^	9.41 ± 1.04^a^	7.94 ± 0.64^a^	9.89 ± 1.76^a^	1.70	0.245
Leucine	8.85 ± 0.33^a^	8.86 ± 0.33^a^	8.47 ± 0.11^a^	9.03 ± 1.04^a^	0.50	0.694
Isoleucine	3.77 ± 0.11^a^	4.97 ± 0.25^b^	4.00 ± 0.22^a^	4.12 ± 0.53^a^	8.24	0.008
Histidine	3.19 ± 0.68^b^	2.00 ± 0.34^ab^	1.24 ± 0.05^a^	3.08 ± 0.74^b^	9.22	0.006
Alanine	2.77 ± 0.05^a^	3.39 ± 0.07^b^	3.42 ± 0.03^b^	3.05 ± 0.27 ^ab^	13.60	0.002
Arginine	3.14 ± 0.47^ab^	3.35 ± 0.56^ab^	2.65 ± 0.32^a^	4.20 ± 0.84^b^	3.74	0.060
Glutamic acid	2.97 ± 0.19^a^	2.84 ± 0.12^a^	2.69 ± 0.15^a^	2.70 ± 0.41^a^	0.87	0.497
Glycine	12.31 ± 0.97^a^	12.72 ± 2.87^a^	12.40 ± 1.03^a^	14.83 ± 1.95^a^	1.21	0.369
Proline	8.71 ± 0.17^a^	9.85 ± 0.51^a^	8.94 ± 0.15^a^	9.19 ± 1.10^a^	1.92	0.205
Tyrosine	2.33 ± 0.07^a^	2.83 ± 0.24^b^	2.21 ± 0.02^a^	2.40 ± 0.29^ab^	6.09	0.018
TAA	81.92	83.93	79.46	87.65		
TEAA	49.69	48.95	47.14	51.30		
NEAA	32.23	34.98	32.32	36.35		
%(TEAA/TAA)	60.65	58.32	59.33	58.53		
TEAA/NEAA	1.54	1.40	1.46	1.41		

All values are expressed as mean ± Standard Deviation (SD). Means with different superscripts within each row are significantly different (*p*< 0.05) as determined by the post-hoc Tukey’s Honestly Significant Difference (HSD).

The percent TEAA/TAA and the ratio of EAA/NEAA of our experimental insects ranged from 58.32–60.65% and 1.40–1.54 respectively. The WHO recommends that for a good quality protein, the ratio of EAA to TAA and EAA to NEAA should be at least 0.4 and 0.6 respectively [55]. Herein, we report results that exceeds the WHO thresholds suggesting that the two insects could be used as sources of good quality protein.

**Fatty acids profile.** Fatty acids are substrates for muscular contraction and general body metabolism. In this study, fatty acids assessment revealed 43 fatty acids with saturated fatty acids (SFAs), monounsaturated fatty acids (MUFAs) and polyunsaturated fatty acids (PUFAs) accounting for 40.83–82.35%, 17.20–57.45% and 0.41–1.74%, respectively, of the total fatty acids detected in oils extracted from the two sets of beetle larvae per location (**Table 2**). These fatty acids ratios (except for *T*. *jaspideus* acquired from Mau forest) corroborated the findings of Bophimai & Siri [56] on six edible dung beetles, Okaraonye & Ikewuchi [57] on *Oryctes rhinoceros* larvae and Kavle et al. [26] on edible *Prionoplus reticularis* larvae. The dominating proportions of total SFAs and MUFAs are unsurprising in the experimental beetle larvae as they have been previously deduced to account for more than 80% of all fats [12]. The fact that MUFAs are considered health promoting agents suggests that these beetles can make healthy dietary candidates. Methyl tetradecanoate, methyl hexadecanoate and methyl octadecenoate of the SFAs, methyl-9*Z*-hexadecenoate and methyl-9*Z*-octadecenoate of the MUFAs and methyl-5*Z*,8*Z*,11*Z*,14*Z*-eicosatetraenoate of the PUFAs were the predominant and abundant fatty acids detected. These SFAs and MUFAs have vastly been reported as the predominant fatty acids in *Oryctes rhinoceros* larvea [56], dung beetles [57], longhorned beetle larvae [24] and edible beetle *Holotrichia parallela* [43]. Methyl tetradecanoate, methyl hexadecanoate and methyl octadecenoate have been reported to strongly associate with characteristic flavours of food products [58]. However, their levels, together with other SFAs except methyl octadecanoate, are known to correlate with hypocholesterolaemia occurrences due to associated high levels of low density lipoprotein-cholesterol (LDL-C) [26]. Methyl octadecanoate reportedly suppresses hypercholesterolemia [59], which could typify the beetle larvae as healthy diets. The conspicuously higher amounts of methyl-9*Z*-octadecenoate indicates that *P*. *punctiger* and *T*. *jaspideus* can make healthier dietary sources with the potential of suppressing thrombogenic, cardiovascular, cancer and inflammatory conditions [60]. Consistent reporting of copious levels of methyl-9Z-octadecenoate in insects may be hypothesized to emanate from their indispensable intricate metabolic roles in maintaining fluidity of cell membranes [58]. The current study only detected methyl-5*Z*,8*Z*,11*Z*,14*Z*-eicosatetraenoate as the only omega 6 fatty acids with no omega 3, despite of other studies reporting linoleic (omega 6) and linolenic acids (omega 3) as the most prevalent PUFAs in several insects [12]. The fatty acids spectra and other chemical components of edible insects exists principally as a function of their dietary sources, ecotypes and specific differences [52], hence explaining the detection and non-detection of certain fatty acids as well as the significant inter specific and site-modulated intraspecific variabilities apparent in **Table 2**. Nonetheless, methyl-5*Z*,8*Z*,11*Z*,14*Z*-eicosatetraenoate has been reported to confer fluidity and flexibility to cell membranes, key to the functionalities of all cells, particularly in nervous system, skeletal muscle, and immune system [61]. PUFA/SFA ratio is an index crucial in the assessment of the nutritional quality of dietary lipids. An ideal ratio >0.4 has been recommended by World Health Organization (WHO) to signify cardiovascular healthy diets [62]. The ratios from the two beetle species collected from Kakamega and Mau forests were lower than the reference ratio but agreed with the ratios of 0.07–0.09 reported by Kavle et al. [26] on edible larvae of *Prionoplus reticularis* beetle.

**Table 2:** Fatty acid spectra (μg /g dry matter) of *P*. *punctiger* and *T*. *jaspideus* sampled from Kakamega and Mau forests.

RT	IUPAC name	ω-n (Δn)	Kakamega forest		Mau Forest		df	F-value	P-value
			*P*. *punctiger*	*T*. *jaspideus*	*P*. *punctiger*	*T*. *jaspideus*			
16.16	Methyl undecanoate	C11:0	18.63 ± 9.33	ND	ND	ND			
18.49	Methyl dodecanoate	C12:0	134.19 ± 10.67^c^	38.14 ± 1.27^a^	107.66 ± 9.64^b^	105.83 ± 4.38^b^	3,8	176.32	1.21E-07
18.93	Methyl-3-methylhexadecanoate	C16:0	ND	ND	ND	6.58 ± 0.96			
18.96	Methyl-2,6-dimethyloctanoate	iso-dimethyl-C:8	ND	ND	6.80 ± 0.30	ND			
19.09	Methyl-3,7,11-trimethyl-dodecanoate	iso-trimethyl-C12:0	ND	1.76 ± 0.16	ND	ND			
19.67	Methyl tridecanoate	C13:0	ND	ND	11.49 ± 0.44^b^	4.46 ± 0.60^a^	1,4	266.36	8.25E-05
20.32	Methyl tetradecanoate	C14:0	1033.02 ± 11.73^d^	202.71 ± 0.42^a^	622.62 ± 35.34^b^	908.13 ± 3.30^c^	3,8	1161.2	6.78E-11
20.35	Methyl-12-methyltridecanoate	isomethyl-C13:0	ND	ND	ND	4.67 ± 0.10			
21.51	Methyl-9-methyltetradecanoate	iso-methyl-C14:0	ND	ND	119.75 ± 10.41	ND			
21.87	Methyl decanoate	C10:0	36.73 ± 12.63^d^	1.48 ± 0.37^a^	11.18 ± 1.05^c^	7.38 ± 0.30^b^	3,8	528.6	1.56E-09
22.18	Methyl-3,7,11-trimethyldodecanoate	iso-trimethyl-C12:0	ND	ND	ND	6.41 ± 0.12			
22.92	Methyl hexadecanoate	C16:0	3041.84 ± 13.23^d^	962.06 ± 31.52^a^	1449.93 ± 6.12^b^	2732.94 ± 44.80^c^	3,8	2234.3	4.97E-12
23.21	Methyl-2-methylhexadecanoate	iso-methyl-C16:0	ND	ND	ND	17.06 ± 0.68			
23.30	Methyl-10-methylhexadecanoate	iso-methyl-C16:0	55.45 ± 13.45	ND	ND	ND			
23.23	Methyl-5,9,13-trimethyltetradecanoate	iso-trimethyl-C14:0	ND	ND	49.55 ± 0.46	ND			
24.82	Methyl octadecanoate	C18:0	34.49 ± 14.33^a^	346.22 ± 9.62^b^	407.30 ± 2.85^c^	30.31 ± 0.19^a^	3,8	4779.8	2.38E-13
26.55	Methyl-18-methylnonadecanoate	isomethyl-C19:0	ND	36.70 ± 0.41					
25.59	Methyl nonadecanoate	C19:0	ND	13.66 ± 0.17					
27.36	Methyl heicosanoate	C21:0	ND	ND	24.73 ± 0.42				
27.67	Methyl-11-methyloctadecanoate	isomethyl-C17:0	ND	ND	ND	13.78 ± 0.12			
28.14	Methyl docosanoate	C22:0	42.90 ± 16.25^b^	ND	43.70 ± 0.12^c^	13.61 ± 0.14^a^	2,6	10979	2.04E-11
28.90	Methyl tricosanoate	C23:0	33.44 ± 16.69^b^	ND	ND	18.98 ± 0.20^a^	1,4	1150.1	4.51E-06
29.62	Methyl tetracosanoate	C24:0	59.87 ± 17.10^b^	ND	ND	30.35 ± 0.20^a^	1,4	19756	1.54E-08
31.34	Methyl hexacosanoate	C26:0	22.98 ± 18.10	ND	ND	ND			
	**∑SFA**		**4513.54**	**1602.73**	**2854.71**	**3900.49**			
20.46	Methyl-11*Z*-tetradecenoate	C14:1 (n-3)	ND	ND	94.07 ± 3.88	ND			
20.59	Methyl-9E-tetradecenoate	C14:1 (n-5)	57.60 ± 11.89	ND	ND	ND			
21.64	Methyl-7*Z*-hexadecenoate	C16:1 (n-9)	ND	ND	31.45 ± 6.37	ND			
21.87	Methyl-5*Z*-dodecenoate	C12:1 (n-7)	27.03 ± 12.50^b^	2.60 ± 0.20^a^	40.54 ± 5.87^c^	ND	2,6	88.929	3.48E-05
22.25	Methyl-9*E*-14-methylpentadecenoate	iso-methyl-C15:1 (n-6)	ND	ND	14.26 ± 0.66	ND			
22.70	Methyl-9*Z*-hexadecenoate	C16:1 (n-7)	843.87 ± 13.10^d^	144.23 ± 1.32^a^	500.95 ± 1.77^b^	555.53 ± 9.31^c^	3,8	1092.4	8.64E-11
23.59	Methyl-9*Z*-heptadecenoate	C17:1 (n-8)	ND	ND	160.87 ± 0.28	ND			
23.62	Methyl-8*Z*-heptadecenoate	C17:1 (n-9)	ND	24.44 ± 0.34	ND	ND			
23.64	Methyl-10*Z*-heptadecenoate	C17:1 (n-7)	370.10 ± 13.65^c^	77.78 ± 0.31^a^	ND	190.08 ± 4.22^b^	2,6	3920.2	4.47E-10
24.06	Methyl-6*E*-octadecenoate	C17:1 (n-12)	ND	ND	ND	9.24 ± 0.38			
24.18	Methyl-7*E*-octadecenoate	C18:1 (n-11)	ND	ND	ND	14.82 ± 0.11			
24.27	Methyl-13*Z*-octadecenoate	C18:1 (n-5)	ND	12.56 ± 0.26	ND	ND			
24.53	Methyl-11*Z*-octadecenoate	C18:1 (n-7)	95.11 ± 14.16	ND	ND	ND			
24.60	Methyl-9*E*-octadecenoate	C18:1 (n-9)	ND	ND	2582.78 ± 6.12^b^	25.32 ± 0.39^a^	1,4	521619	2.21E-11
24.62	Methyl-9*Z*-octadecenoate	C18:1 (n-9)	4861.04 ± 14.20^d^	30.16 ± 0.12^a^	590.57 ± 10.06^b^	4445.73 ± 71.52^c^	3,8	14298	2.98E-15
25.47	Methyl-10*Z*-nonadecenoate	C19:1 (n-9)	72.99 ± 14.71^b^	42.90 ± 0.50^a^	ND	ND	1,4	171.43	0.000197
	**∑MUFA**		**6327.73**	**334.67**	**4015.49**	**5240.72**			
24.27	Methyl-11*E*,14*E*-octadecadienoate	C18:2 (n-4)	ND	ND	11.06 ± 1.56	ND			
25.99	Methyl-5*Z*,8*Z*,11*Z*,14*Z*-eicosatetraenoate	C20:4 (n-6)	172.72 ± 15.01^c^	ND	110.29 ± 0.83^b^	24.98 ± 0.28^a^	2,6	1506.1	7.86E-09
26.12	Methyl-7*E*,10*E*,13*E*-icosatrienoate	C20:3 (n-7)	ND	8.73 ± 0.25^a^	ND	12.70 ± 0.39^b^	1,4	222.23	0.000118
	**∑PUFA**		**172.72**	**8.73**	**121.35**	**37.68**			
	PUFA/SFA		0.04	0.01	0.04	0.01			

All values are expressed as mean ± Standard Deviation (SD); n = 3; ND—not detected; Means with different superscript small letters within each row are significantly different (p*<*0.05), as determined by the post-hoc Tukey’s Honestly Significant Difference (HSD) test. SFA-Saturated Fatty Acids; MUFA-Monounsaturated Fatty Acid; PUFA-Polyunsaturated Fatty Acids.

#### Entomochemical contents

The total flavonoids (TFC) of beetles ranged between 3.6 to 6.9 mg QE/g. In both the sampling sites, *T*. *jaspideus* had significantly lower TFC than *P*. *punctiger* (**Table 3**). The total phenol (TPC) contents ranged between 4.4 to 7.8 mg GAE/g, comparable to that of the ground cricket (*Henicus whellani*) (7.7 mg GAE/g) [63] and significantly more than that for uncooked *Eulepida mashona* (0.81 mg GAE/g dw) [64]. Likewise to TFC, TPC of *T*. *jaspideus* was significantly lower than TPC of *P*. *punctiger* in both the collection sites.

**Table 3:** Total phenolic and flavonoid contents of *T*. *jaspideus* and *P*. *punctiger* collected from Kakamega and Mau forests.

Parameters	*P*. *punctiger*		*T*. *jaspideus*	
	Kakamega Forest	Mau Forest	Kakamega Forest	Mau Forest
Total phenols (mg GAE/g)	7.81 ± 0.208^d^	7.10 ± 0.230^c^	6.44 ± 0.303^b^	4.42 ± 0.211^a^
Total flavonoids (mg QE/g)	6.88 ± 0.206^c^	6.19 ± 0.673^b^	5.34 ± 0.277^bc^	3.58 ± 0.068^a^

Values are expressed as mean ± standard deviation (SD); n = 3. Means with different superscript small letters within each row are significantly different (p<0.05)

As previously determined that insects sequester phenolic compounds from their diet [65], the insects herein may not be exceptional and therefore the differences in TPC and TFC observed between the sampling sites could be attributed to possible differences in concentration of phenolic compounds in dead logs they inhabited. For example, *Prunus africana*, one of the predominant wood variety inhabited by the larvae in Kakamega forest has been shown to be rich in TPC (55.14 mg/g dw) [66]. Lower concentration ranges of TPC and TFC (8.02–15.52 mg GAE/g dw) and (3.42–7.45 mg QE/g), respectively, has been reported for *Croton macrostachyus* [67], one of the predominant wood variety inhabited by sample larvae in Mau forest. Although the occurrence of phenolics in insects is strongly linked to their diet, they are also able to synthesize non-dietary phenolics through enzyme mediated reaction, termed sclerotization [68]. A study by Hirayama et al. [69] isolated two phenolic compounds: quercetin 3-*O-β*-D-galactopyranosyl-(1→3)-*β*-D-galactopyranoside and kaempferol 3-*O*-*β*-D-galactopyranosyl-(1→3)-*β*-D-galactopyranoside from the cocoon of *Rondotia menciana* fed exclusively on mulberry leaves. The two compounds were not detected in the mulberry leaves, positioning insects as a potential novel source of important phenolic compounds that cannot be synthesized by plants. The presence of phenolic compounds within edible insects has become a subject of research concerning their nutritional roles. Additionally, their potential to impart colour and flavour in prepared dishes enhances their value, rendering them noteworthy subjects for research within the food industry [19]. Phenolic compounds have long been recognized to have anti-inflammatory, antimicrobial, anticancer and antioxidant properties [70].

#### Antioxidant activities

**Fig 3:**
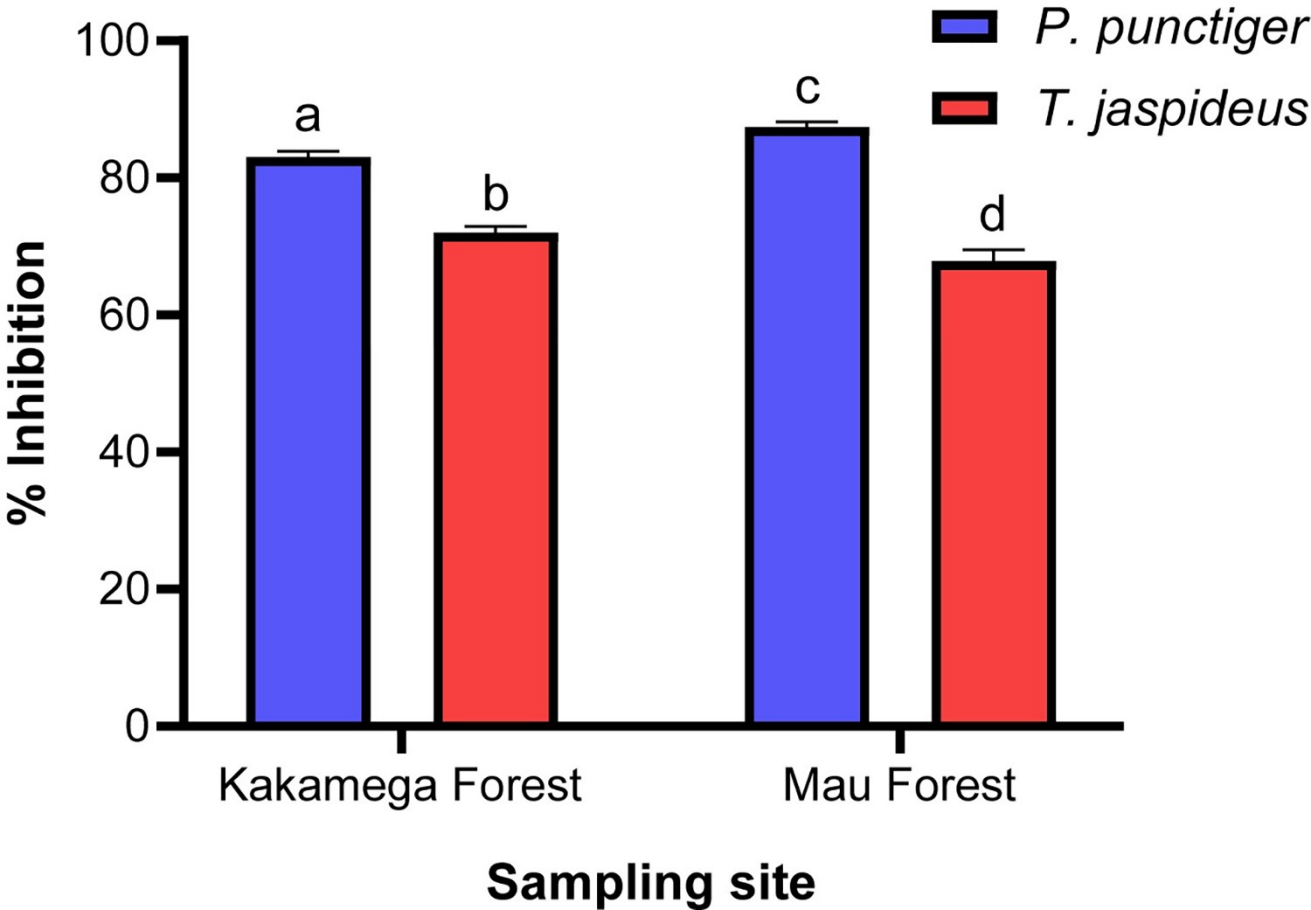
Radical scavenging activity of *T*. *jaspideus* and *P*. *punctiger*. Values are percentage means ± standard deviation of three independent experiments. Means with different letters are statistically different (*p*< 0.05).

The DPPH radical-scavenging assay, herein adopted, is a widely used method to evaluate the antioxidant activity of natural compounds, plant extracts, and food matrices [71]. The free radical scavenging activities of the insect powder extracts (2.5 mg/mL MeOH) ranged between 67% - 87% (Fig 3). The beetle larva *P*. *punctiger* collected from Mau forest exhibited the highest antioxidant activity (87.43%), whereas *T*. *jaspideus* collected from the same forest had the least antioxidant activity (67.87%). The sampling sites and species portray to have had a significant effect on the antioxidant potential of the extracts of the insects. These results indicated higher levels than that for *Macrotermes subhylanus* (55.57%), *Gonimbrasia belina* (37.44%) and *Hermetia illucens* (3.63%) as determined by [72] at concentrations of 50 mg dw/mL in Milli-Q water. The results are however closely similar to that of *Anaphe panda* (78.2%) hexane extracts (50 mg dw/100 mL hexane) [40]. The high antioxidant activity could be attributed to the high phenols and flavonoids recorded in these samples in this study.

This was concordant with previous studies that elucidated the significant positive correlation between total phenolic content and free radical reducing potentials of the study extract [73–76]. Phenols and polyphenols have high redox potentials making them inherent hydrogen donors and thus their ability to quench free radicals including hydroxyls and super oxides [77, 78]. Insects majorly derive phenolic compounds from the food they eat, however some processes such as sclerotization and melanisation have also been shown to yield these compounds *de-novo* [68, 79]. The beetle larva of *P*. *punctiger* inhabited the superficial surface of a rotting wood and thus majorly fed on the phloem (outer and inner bark), partly cambium and the sapwood whereas *T*. *jaspideus* burrows deep into the heartwood where phenolic compounds are limited. Plant phytochemicals are primarily responsible for defence against aggression by pathogens, pests, parasites, predators and ultraviolet radiations as well as colour and fragrance, hence their heavy accumulation in exposed surfaces [80, 81]. Antioxidants are important in human meal since they play important role in protection against free radicals that otherwise would cause chronic inflammatory diseases such as cardiovascular diseases, aging, anaemia and cancers [82]. Their importance and roles have been widely studied and reviewed [83–87]. The two beetle larvae *P*. *punctiger* and *T*. *jaspideus* can thus be used as dietary supplements for important natural antioxidants.

## Conclusion

This study reports for the first-time the nutritional and entomochemical benefits of the larvae of two wood-boring beetle species in the forested zones of Kenya. The beetles showed appreciable amounts of high-quality protein with adequate levels of cereal limiting essential amino acids particularly lysine, methionine, and others that were above the required standards set by the World Health Organization for good quality food sources. The beetle larvae exhibited rich profiles of beneficial MUFAs and PUFAs characterized by abundant levels of methyl-9*Z*-octadecenoate, and methyl-5*Z*,8*Z*,11*Z*,14*Z*-eicosatetraenoate, respectively. The beetle larvae were also endowed with substantial antioxidant activity, which could contribute to potential health benefits. These findings suggest that *T*. *jaspideus* and *P*. *punctiger* could be considered as a promising alternative source of proteins and nutraceuticals. This implies that both beetle larvae derived products could be used as functional ingredients in food fortification that might be helpful in addressing global food insecurity. Antinutrients, herein not determined, might as well exist, necessitating future studies to determine antinutritional profiles of these wood borers. Further, studies are recommended to determine their full nutritional profile, sensory, and economic feasibility for their integration into the existing food and pharmaceutical industry.
